# HPV vaccination hesitancy and acceptance among parents in Saxony-Anhalt, Germany: the role of gender, awareness and fear

**DOI:** 10.1186/s12889-025-26061-1

**Published:** 2025-12-23

**Authors:** Paolo Gennari, Daniel Schlund, József Mészáros, Atanas Ignatov

**Affiliations:** Department of Gynecology and Obstetrics, Otto-von-Guericke University, Gerhard-Hauptmann-Str. 35, 39108 Magdeburg, Germany

**Keywords:** HPV vaccination, Parental attitudes, Vaccine hesitancy, HPV awareness

## Abstract

**Background:**

Despite national recommendations in Germany for universal HPV vaccination, uptake remains suboptimal—particularly among boys. Parental awareness, gender dynamics, and hesitancy play key roles in vaccination decisions.

**Methods:**

A cross-sectional survey was conducted among 406 parents of children aged 9–14 years in Magdeburg, Saxony-Anhalt. An anonymized online questionnaire assessed HPV-associated awareness, attitudes, willingness to vaccinate, and conditions under which hesitant parents might reconsider. Statistical analyses included chi-square tests and logistic regression. Parent–child gender dyads were examined to explore interaction effects. Vaccine hesitancy was conceptualized along a continuum from acceptance to refusal, although for analysis we contrasted parents willing to vaccinate with those refusing vaccination.

**Results:**

Mothers were significantly more aware of HPV than fathers (86.8% vs. 68.0%; *p* < 0.001) and more willing to vaccinate their children (90.4% vs. 74.8%; *p* < 0.001). Parents of daughters expressed greater fear of HPV-associated disease than parents of sons (67.5% vs. 37.4%; *p* < 0.001), although willingness to vaccinate did not differ by child gender. Dyadic analysis highlighted gender-specific patterns, with father–daughter pairs reporting high perceived disease risk yet comparatively lower willingness to vaccinate. Father–son pairs showed the lowest awareness and engagement overall. Logistic regression identified three determinants of willingness to vaccinate: prior awareness of HPV (OR = 3.77; *p* = 0.006), fear of disease (OR = 3.69; *p* = 0.006), and higher education (OR = 1.60; *p* = 0.032). Among parents who refused HPV vaccination, 80% reported that their decision would not change under any of the proposed conditions.

**Conclusions:**

Gender disparities in HPV vaccine awareness and acceptance persist. Emphasizing the role of male vaccination in reducing transmission and protecting both sexes could improve uptake. Targeted strategies should strengthen engagement of all parents, with particular efforts to involve fathers and correct gendered misperceptions.

**Supplementary Information:**

The online version contains supplementary material available at 10.1186/s12889-025-26061-1.

## Introduction

Human papillomavirus (HPV) is one of the most common sexually transmitted infections worldwide and is causally linked to several types of cancer, including cervical, anal, penile, and oropharyngeal malignancies. Persistent infection with high-risk HPV types is responsible for nearly all cases of cervical cancer, making prevention through vaccination a critical public health priority [1].

In Germany, HPV vaccination has been recommended by the Standing Committee on Vaccination (STIKO) since 2007 for girls and since 2018 also for boys, ideally between the ages of 9 and 14. Despite this, national uptake remains suboptimal: in 2021, only 52% of 15-year-old girls and 27% of boys had completed the HPV vaccination series, falling short of WHO’s goal of 90% coverage for cervical cancer elimination [2]. In Saxony-Anhalt, vaccination coverage is even lower, with less than 45% of girls and fewer than 20% of boys completing the series (Robert Koch Institute, 2022; Saxony-Anhalt Regional Health Authority, 2023). These figures underline the challenge of moderate uptake in this region.

Although HPV vaccination is recommended nationally, implementation strategies vary across federal states and there is no unified school-based delivery system. Regional and structural context may therefore influence vaccination practices. Previous public health monitoring in Germany has documented regional differences in vaccination behaviour: for example, studies during the COVID-19 vaccination campaign found lower uptake among residents of rural areas and in eastern federal states compared with urban and western regions, suggesting that access barriers, health system trust, and socio-demographic composition may play a role [3, 4]. While comparable data for HPV vaccination specifically are limited, these patterns indicate that contextual and structural factors may also contribute to the comparatively low HPV vaccination uptake observed in Saxony-Anhalt.

In recent years, international research has highlighted substantial variation in HPV vaccination uptake, parental acceptance, and hesitancy across countries, settings, and population sub-groups. A recent systematic review of 86 studies found that, while many parents support HPV vaccinations for their children, acceptance rates vary widely—ranging from very low in some settings to quite high in others—with key influencing factors including parental knowledge, perceived safety, cost, and healthcare provider recommendation [5]. Within Europe, a 2024 meta-analysis shows that parents’ hesitancy is associated with socio-demographic features (such as immigrant status, employment status, religion), psychological factors such as risk perception and safety concerns, as well as communication and awareness [6]. Globally, interventions to increase uptake (beyond just awareness raising) — including school-based vaccination programs, multicomponent and systems-level strategies, provider reminders, and decision support — have proved more effective [7–9]. These findings suggest that any study of HPV vaccine uptake (or intent to vaccinate) needs to account for not only the immediate local context (policies, cost, access), but also the broader social, economic, and communication environment, since those international patterns may help explain observed similarities or differences.

Parental attitudes and beliefs are among the most decisive determinants of HPV vaccination uptake in minors. Prior studies have shown that awareness of HPV and its consequences, trust in healthcare providers, fear of side effects, and sociocultural perceptions of sexuality significantly shape vaccination intentions. Moreover, gender norms and role expectations influence both parental engagement and vaccine acceptance. For instance, fathers are generally less informed about HPV and less involved in vaccination decisions, particularly for daughters [10–13].

While much of the existing literature originates from Anglo-American or pan-European contexts, local cultural factors may substantially affect attitudes toward vaccination. To our knowledge, no recent study has systematically assessed HPV-associated awareness and hesitancy among German parents since the expansion of vaccination recommendations to include boys. Furthermore, dyadic analyses that explore the interaction between the gender of the parent and the child are lacking, despite their relevance for communication strategies and public health outreach. Unlike most prior studies, which analyze parents in aggregate, our study applied a dyadic approach to examine how parent–child gender combinations influence HPV vaccination attitudes. This perspective has not yet been systematically explored in Germany and provides new insight into local drivers of vaccine hesitancy.

Beyond socio-demographic and contextual factors, contemporary models of vaccine behaviour emphasize psychological antecedents such as confidence, complacency, constraints, and social norms. The 7 C model of vaccine acceptance, validated among parents and adolescents in Europe, summarises these determinants as confidence, complacency, constraints, calculation, collective responsibility, confidence in the health system, and social conformity [14, 15]. Constructs examined in our study, such as HPV awareness, fear of HPV-associated disease, and educational level, can be mapped onto these dimensions, for example awareness and fear reflecting confidence and perceived disease threat, and education relating to calculation and access to trustworthy information. Positioning our findings within this framework facilitates comparison with international vaccine hesitancy research and highlights modifiable targets for intervention.

This study aimed to assess HPV-associated awareness, attitudes, and willingness to vaccinate among parents of school-aged children (aged 9–14 years) in Magdeburg, Saxony-Anhalt. We investigated differences by parent and child gender, explored parent–child dyad patterns, identified determinants of vaccination willingness, and examined conditional factors under which hesitant parents might reconsider. By capturing local attitudes in a region with moderate uptake, this study aims to inform targeted interventions to improve HPV vaccination coverage in Saxony-Anhalt.

## Materials and methods

### Study design and participants

This cross-sectional study was conducted in Magdeburg, Germany, during the first quarter of 2025. The target population consisted of parents or legal guardians of children aged 9–14 years, corresponding to the recommended age range for HPV vaccination under German guidelines. Participants were recruited via a digital, anonymized questionnaire (see Supplementary File 1) distributed through four middle schools in Magdeburg. A total of 12 schools in the Magdeburg municipal area were contacted and invited to support the study by distributing the survey link to parents. Four schools agreed to participate and shared the link via their existing communication platforms. Schools declining participation cited limited administrative capacity or internal data privacy concerns. No objections regarding the study content were reported. Within these schools, approximately 1195 eligible students were enrolled in the relevant age group, and 406 parents provided complete responses, yielding an estimated response rate of 33.9%. Only fully completed questionnaires were included in the analysis. Partially completed surveys and responses with missing data on the main outcome were excluded, and no imputation of missing values was performed. Before accessing the survey, parents viewed an electronic consent statement outlining study aims, anonymity, and voluntary participation. Consent was provided by selecting an ‘I agree’ checkbox, which was required to proceed. Inclusion criteria were: [1] being the parent or legal guardian of a child aged 9–14 years; [2] residing in the Magdeburg area; and [3] provision of informed consent for anonymous participation.

### Questionnaire and variables

The questionnaire comprised five sections. The first section collected sociodemographic characteristics, including the parent’s age, gender, marital status, education level, occupation, and annual household income. The second section focused on child characteristics, specifically age and gender. The third section assessed HPV awareness by asking whether the parent had heard of HPV or HPV-associated diseases. The fourth section addressed HPV vaccination awareness and attitudes, including whether the parent had heard of the HPV vaccine, actively sought information, and whether they were willing to vaccinate their child. The fifth section explored barriers and conditions for acceptance through multiple-response items assessing which circumstances might persuade vaccine-hesitant parents to accept HPV vaccination.

HPV vaccination status of the child was self-reported. The main outcome variable, willingness to vaccinate, was defined as answering “yes” to the question “Would you have your child vaccinated against HPV?”. In line with contemporary literature, vaccine hesitancy is understood as a position on a continuum between full acceptance and refusal, including indecision and delay. In the present analysis, we contrasted parents who were willing to vaccinate with those who answered “no” to this question, whom we classified as refusing HPV vaccination. We therefore refer to this subgroup as ‘refusing’ in a strict behavioural sense, while acknowledging that broader conceptualisations of hesitancy also encompass more ambivalent positions. For analytical purposes, parent–child gender dyads (e.g., mother–daughter, father–son) were constructed to explore interaction effects.

The questionnaire was developed specifically for this study, with items adapted from previously validated instruments in European studies on parental vaccine hesitancy and HPV vaccination [10–13]. The questionnaire underwent cognitive pre-testing with five parents from the target population to assess clarity and comprehension. Minor wording adjustments were made based on feedback. Given that most items were adapted from validated European instruments, no additional psychometric validation (e.g., Cronbach’s alpha) was conducted. An English version of the full questionnaire is provided as Supplementary File 1.

No a priori power calculation was performed, as recruitment feasibility depended on school participation. However, the final sample exceeded minimum recommended thresholds for logistic regression modelling based on events-per-variable guidelines.

### Statistical analysis

Descriptive statistics were calculated to characterize the sample. Pearson’s chi-square test was used to assess associations between sociodemographic variables and awareness or attitudes. Variables were entered into the logistic regression model based on (a) significance in bivariate analysis (*p* < 0.10) and (b) theoretical relevance according to established vaccine hesitancy research frameworks. Multicollinearity was assessed using variance inflation factors (VIF), and no variable exceeded acceptable thresholds (VIF < 2.5). Model performance was evaluated using the area under the receiver operating characteristic (ROC) curve (AUC).

All analyses were performed using IBM SPSS Statistics for Windows, Version 28.0 (IBM Corp., Armonk, NY), with statistical significance defined as *p* < 0.05.

## Results

### Sample characteristics

A total of 406 parents of school-aged children (aged 9–14 years) in Magdeburg, Germany, completed the questionnaire (Table 1). The sample was predominantly female (74.6%), with a mean age of 40.9 years (SD = 6.3). Children were evenly split by gender. Most respondents were married (54.8%) and had completed vocational training or a university degree (65.1%). The most common employment sectors were healthcare/social services (35.1%) and office/administration (29.7%). Annual household income varied, with 26.5% earning €40,000–60,000 per year.

**Table 1:** Demographic characteristics of parents of school-aged children in Magdeburg, Germany

Characteristic	Frequency (*n*)	Percent (%)
Age of parents (mean ± SD)	–	40.9 ± 6.3
Gender of parents		
Female	303	74.6
Male	103	25.4
Age of child (years)	–	9–14 (modal age = 12)
Gender of child		
Female	203	50.0
Male	203	50.0
Marital status		
Married	223	54.8
In a registered partnership	58	14.3
Single	61	15.0
Divorced	41	10.1
Widowed	23	5.7
Highest educational qualification		
Lower secondary education	29	7.1
Intermediate secondary education	42	10.3
University entrance qualification	67	16.5
Vocational training or university degree	265	65.1
No response	3	0.7
Employment sector		
Industrial/production work	39	9.6
Service sector	71	17.4
Healthcare/social services	143	35.1
Office/administration	121	29.7
Unemployed	32	7.9
Annual gross income (€)		
≤ 20,000	44	10.8
20,000–40,000	99	24.3
40,000–60,000	108	26.5
60,000–100,000	96	23.6
> 100,000	31	7.6
No response	28	6.9

### Awareness and attitudes by parent gender

Mothers were significantly more likely than fathers to have heard of HPV (86.8% vs. 68.0%; *p* < 0.001) and of the HPV vaccine (96.4% vs. 89.3%; *p* = 0.006), and to have actively sought information (68.3% vs. 44.7%; *p* < 0.001). Willingness to vaccinate was higher among mothers than fathers (90.4% vs. 74.8%; *p* < 0.001). Mothers were also more likely to agree that HPV vaccination should occur before first sexual intercourse (86.8% vs. 62.1%; *p* < 0.001) and to prefer vaccination at 9–11 years (63.7% vs. 39.8%; *p* < 0.001) (Table 2).

**Table 2:** HPV-associated awareness and attitudes by gender of parent and child (Magdeburg)

Question	Parent Female (*N* = 303)	Parent Male (*N* = 103)	*p*-value		child female (*N* = 203)	child male (*N* = 203)	*p*-value
Have you heard of HPV?							
Yes	263 (86.8%)	70 (68.0%)	< 0.001		168 (82.8%)	165 (81.2%)	0.698
No	40 (13.2%)	33 (32.0%)			35 (17.2%)	38 (18.7%)	
Have you heard of the HPV vaccine?							
Yes	292 (96.4%)	92 (89.3%)	0.006		192 (94.6%)	192 (94.6%)	1.000
No	11 (3.6%)	11 (10.7%)			11 (5.4%)	11 (5.4%)	
Have you actively sought information about HPV or the vaccine?							
Yes	207 (68.3%)	46 (44.7%)	< 0.001		136 (67.0%)	117 (57.6%)	0.124
No	85 (28.1%)	46 (44.7%)			56 (27.6%)	75 (36.9%)	
Would you have your child vaccinated against HPV?							
Yes	279 (90.4%)	77 (74.8%)	< 0.001		176 (86.7%)	175 (86.2%)	0.885
No	29 (9.6%)	26 (25.2%)			27 (13.3%)	28 (13.8%)	
When should HPV vaccination be given to children?							
before first sexual intercourse	263 (86.8%)	64 (62.1%)	< 0.001		168 (82.8%)	159 (78.3%)	0.231
after first sexual intercourse	0 (0%)	0 (0%)			0 (0%)	0 (0%)	
do not know	34 (19.9%)	30 (31.6%)			8 (3.9%)	16 (7.9%)	
Preferred age for HPV vaccination							
< 9	6 (2.0%)	5 (2.8%)	< 0.001		6 (3.0%)	41 (39.8%)	0.092
9–11	193 (63.7%)	41 (39.8%)			130 (64.0%)	17 (16.5%)	
12–14	61 (20.1%)	17 (16.5%)			29 (14.3%)	1 (1.0%)	
> 15	2 (0.7%)	1 (1.0%)			1 (0.5%)	13 (12.6%)	
do not know	12 (4.0%)	13 (12.6%)			10 (4.9%)	15 (7.4%)	
Are you afraid of your child developing an HPV-associated disease?							
Yes	167 (78.4%)	119 (71.3%)	0.151		137 (67.5%)	76 (37.4%)	< 0.001
No	46 (21.6%)	48 (28.7%)			57 (28.1%)	110 (54.2%)	

## Awareness and attitudes by child gender

Parental awareness of HPV (82.8% vs. 81.2%; *p* = 0.698), awareness of the HPV vaccine (94.6% vs. 94.6%; *p* = 1.000), active information seeking (67.0% vs. 57.6%; *p* = 0.124), and willingness to vaccinate (86.7% vs. 86.2%; *p* = 0.885) did not differ significantly between parents of daughters and parents of sons. However, parents of daughters were significantly more likely to report fear of their child developing an HPV-associated disease (67.5% vs. 37.4%; *p* < 0.001) (Table 2).

## Parent–Child gender dyads

Gender combinations of parent–child dyads revealed notable differences in awareness and attitudes (Table 3). Mothers of daughters exhibited the highest HPV awareness (86.5%) and willingness to vaccinate (91.7%). Fathers of daughters, in contrast, reported high fear of HPV-associated disease (74.5%) but lower willingness to vaccinate (70.2%). Fathers of sons consistently showed the lowest HPV awareness (66.1%) and comparatively low willingness (78.6%). Most comparisons across dyads reached statistical significance. The only exception was awareness of HPV-associated diseases, where differences between dyads did not reach significance (*p* = 0.293).

**Table 3:** – Parent–Child gender dyads and attitudes toward HPV and vaccination (*N* = 406)

Item	Mother–Daughter (*N* = 156)	Mother–Son (*N* = 147)	Father–Daughter (*N* = 47)	Father–Son (*N* = 56)	*p*-value
Heard of HPV					
Yes	135 (86.5%)	128 (87.1%)	33 (70.2%)	37 (66.1%)	< 0.001
No	21 (13.5%)	19 (12.9%)	14 (29.8%)	19 (33.9%)	
Heard of HPV-associated disease					
Yes	150 (96.2%)	136 (92.5%)	44 (93.6%)	50 (89.3%)	0.293
No	6 (3.8%)	11 (7.5%)	3 (6.4%)	6 (10.7%)	
Fear of HPV-associated disease					
Yes	102 (65.4%)	65 (44.2%)	35 (74.5%)	11 (19.6%)	< 0.001
No	48 (30.8%)	71 (48.3%)	9 (19.1%)	39 (69.6%)	
Don’t know	6 (3.8%)	11 (7.5%)	3 (6.4%)	6 (10.7%)	
Heard of HPV vaccine					
Yes	148 (94.9%)	144 (98.0%)	44 (93.6%)	48 (85.7%)	0.007
No	8 (5.1%)	3 (2.0%)	3 (6.4%)	8 (14.3%)	
Actively sought information					
Yes	110 (70.5%)	97 (66.0%)	26 (55.3%)	20 (35.7%)	< 0.001
No	38 (24.4%)	47 (32.0%)	18 (38.3%)	28 (50.0%)	
Don’t know	8 (5.1%)	3 (2.0%)	3 (6.4%)	8 (14.3%)	
Willing to vaccinate child					
Yes	143 (91.7%)	131 (89.1%)	33 (70.2%)	44 (78.6%)	< 0.001
No	13 (8.3%)	16 (10.9%)	14 (29.8%)	12 (21.4%)	

### Predictors of willingness to vaccinate

In logistic regression analysis (Table 4), three determinants of willingness to vaccinate emerged as significant: having heard of HPV (OR = 3.77; 95% CI: 1.48–9.65; *p* = 0.006), reporting fear of HPV-associated disease (OR = 3.69; 95% CI: 1.45–9.41; *p* = 0.006), and higher educational level (OR = 1.60; 95% CI: 1.04–2.46; *p* = 0.032). Parent gender, child gender, active information-seeking behaviour and employment status were entered into the model but were not statistically significant predictors. These results suggest that cognitive and affective factors, rather than demographic characteristics alone, primarily shaped parental intention to vaccinate.

**Table 4:** – Logistic regression analysis: predictors of willingness to vaccinate

Predictor	B	SE	Wald	df	*p*-value	OR (Exp(B))	95% CI (Lower–Upper)
Parent gender (male)	−0.698	0.469	2.212	1	0.137	0.498	0.198–1.248
Child gender (male)	0.710	0.434	2.671	1	0.102	2.034	0.868–4.764
Heard of HPV before study	1.328	0.479	7.676	1	0.006	3.772	1.475–9.648
Fear of disease	1.305	0.478	7.459	1	0.006	3.687	1.445–9.405
Actively sought info	−0.418	0.468	0.799	1	0.371	0.658	0.263–1.647
Education (high)	0.469	0.219	4.579	1	0.032	1.599	1.040–2.457
Employment status	−0.195	0.187	1.093	1	0.296	0.823	0.570–1.186
Constant	−1.995	1.539	1.680	1	0.195	0.136	—

### Model performance

**Fig. 1:**
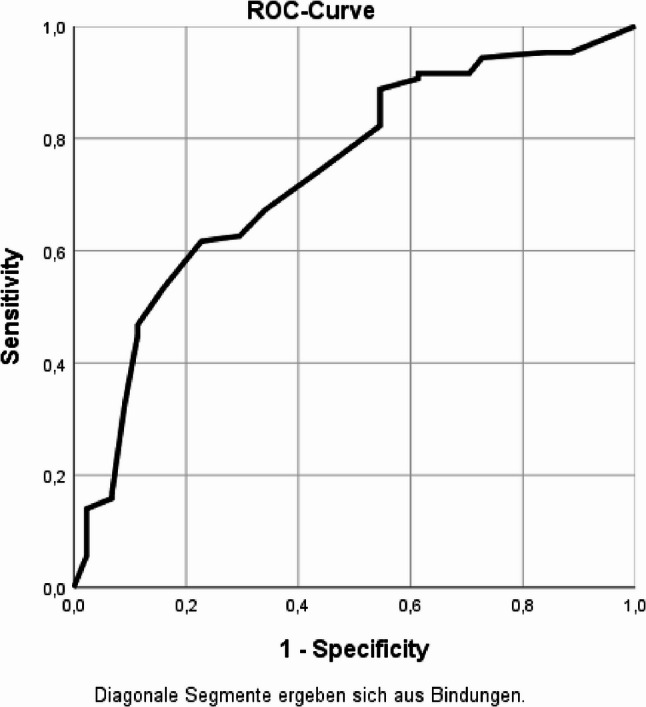
ROC curve for the logistic regression model predicting willingness to vaccinate. AUC = 0.736, indicating acceptable model discrimination

The model showed acceptable fit (Hosmer–Lemeshow *p* = 0.326) and classification accuracy (74.2%). The ROC curve yielded an AUC of 0.736, indicating acceptable discriminatory ability (Fig. 1).

### Conditions for acceptance among hesitant parents

Among the 55 parents who initially declined HPV vaccination for their child, 80.0% indicated that no listed condition would change their decision. A minority reported that they might reconsider if the vaccine were recommended by a pediatrician (12.7%), endorsed by friends or family (12.7%), included in the national immunization schedule (10.9%), or offered free of cost (9.1%). Other factors, such as school recommendation (7.3%) or additional assurances of safety (7.3%), were less frequently cited. Stratification by parent–child dyad revealed no consistent pattern of responsiveness (Table 5).

**Table 5:** Conditions under which Vaccine-Hesitant parents would accept HPV vaccination

Condition	Total (*N* = 55)	Mother–Daughter	Mother–Son	Father–Daughter	Father–Son
If recommended by pediatrician	7 (12.7%)	5	1	0	1
If recommended by school	4 (7.3%)	2	1	0	1
If friends/family recommend	7 (12.7%)	4	1	0	2
If included in national schedule (STIKO)	6 (10.9%)	5	0	0	1
If free of cost	5 (9.1%)	4	1	0	0
If safety is guaranteed	4 (7.3%)	3	0	0	1
None of the above	44 (80.0%)	20	12	7	5

In a follow-up question, hesitant parents were asked to indicate why they would not vaccinate their child. The most frequently selected reasons included concerns about vaccine safety, belief that their child was too young, and the perception that HPV vaccination was unnecessary. These reasons align with international findings indicating safety concerns, low perceived susceptibility, and beliefs about sexual maturity as common determinants of HPV vaccine refusal [11, 16, 17].

## Discussion

This study investigated parental awareness, attitudes, and hesitancy toward HPV vaccination among parents in Magdeburg, Saxony-Anhalt. The analysis provides new insights into gendered differences in vaccine awareness and acceptance, determinants of willingness to vaccinate, and the underlying reasons for persistent hesitancy. International evidence shows that similar patterns are observed across diverse settings. Systematic reviews covering Europe, North America, Asia, and Africa consistently identify parental awareness, perceived risk of HPV-associated disease, and trust in healthcare providers as central drivers of vaccine acceptance [5, 6]. Conversely, concerns about vaccine safety and cultural perceptions of sexuality remain among the most common barriers [17, 18]. By positioning our findings within this global context, we can better understand how local attitudes in Saxony-Anhalt both align with and diverge from international experience.

Recent conceptual models emphasise that vaccine intention is shaped not only by knowledge but also by psychological determinants such as confidence, complacency, perceived constraints, social norms and responsibility. The 7 C model of vaccine acceptance, validated among European parents and adolescents, synthesises these determinants into measurable behavioural domains [14]. Constructs assessed in this study, including HPV awareness, perceived disease severity, and trust in healthcare providers, align with components of this framework. Furthermore, vaccine acceptance is increasingly understood as a continuum rather than a binary decision, ranging from full acceptance to uncertainty, delay, or refusal [15]. The operationalisation used in this study therefore reflects only the most decisive end of this spectrum and may underestimate more ambivalent forms of hesitancy.

### Gender disparities in awareness and willingness

Mothers consistently demonstrated higher awareness of HPV and the HPV vaccine, were more likely to actively seek information, and expressed greater willingness to vaccinate compared to fathers. These differences reflect patterns observed in other high-income countries, where maternal involvement in healthcare and higher health literacy among women contribute to stronger engagement with preventive measures [10–13, 16].

By contrast, fathers were both less aware and less willing to vaccinate, despite similar access to information channels. These findings underline the need for targeted communication strategies that specifically address fathers, who remain an under-engaged group in HPV vaccination campaigns. Comparable gendered patterns have been reported internationally: studies in the UK and US found that mothers were more knowledgeable and proactive in HPV vaccination decisions, while fathers were less engaged, particularly for daughters [5, 12]. In contrast, countries with school-based, gender-neutral vaccination programmes, such as Australia and Scotland, report smaller gender gaps, suggesting that structural approaches can mitigate parental role differences [19, 20].

While willingness to vaccinate did not differ between parents of daughters and sons, parents of daughters reported significantly greater fear of HPV-associated disease. This persistent perception of HPV as a “female-focused” infection remains a barrier to engaging parents of boys. Public health efforts should more clearly communicate that HPV vaccination also protects males against anogenital and oropharyngeal cancers and reduces viral transmission, thus contributing to herd immunity [21].

### Dyadic interactions and parental hesitancy

The parent–child dyad analysis revealed more nuanced gender dynamics. Mother–daughter dyads showed the highest awareness and willingness to vaccinate, while father–son dyads demonstrated the lowest awareness. Strikingly, father–daughter dyads combined high levels of fear of HPV-associated disease with comparatively lower willingness to vaccinate. This pattern may reflect cultural discomfort in discussing sexuality and preventive measures across the father–daughter relationship.

Such findings highlight the importance of tailoring interventions to both parental and child gender roles. Fathers, in particular, should be directly addressed in awareness campaigns, both to strengthen their knowledge and to normalize proactive involvement in vaccination decisions for sons and daughters alike [12, 13].

### Determinants of willingness to vaccinate

Multivariable analysis identified three determinants of willingness to vaccinate: prior awareness of HPV, fear of HPV-associated disease, and higher educational attainment. Demographic characteristics such as parent or child gender were not significant once awareness and fear were accounted for. This suggests that emotional and cognitive factors—knowledge of HPV and perceived disease threat—outweigh structural variables in shaping vaccination intentions. These findings are consistent with international studies demonstrating that prior awareness of HPV, perceived disease threat, and higher education levels are robust predictors of parental willingness across settings [5, 6]. Importantly, provider recommendation has emerged as a particularly influential factor in multiple countries [20, 22], underscoring the global relevance of strengthening physician-parent communication.

These results align with previous studies demonstrating that awareness and perceived susceptibility are strong behavioral drivers of vaccine acceptance [10]. The acceptable discriminatory ability of the model further supports the robustness of these determinants.

### Vaccine hesitancy and conditional acceptance

Among parents refusing vaccination, most reported that none of the listed conditions would influence their decision. This suggests a subgroup with strong pre-existing attitudes, distinct from undecided parents described in international literature as ‘fence-sitters,’ who typically respond to paediatric recommendations, social endorsement, or structural facilitation (Table 5). Conditional acceptance was limited and most commonly associated with pediatrician recommendation (12.7%) or social endorsement by family and peers (12.7%), followed by inclusion in the national immunization schedule (10.9%) and cost removal (9.1%). Other factors, such as school recommendation (7.3%) or assurances of safety (7.3%), were rarely cited (Table 5). International comparisons suggest that our proportion of firmly resistant parents may be higher than in other countries, where conditional acceptance is more common. For instance, systematic reviews show that pediatrician or school recommendations often sway undecided parents [5, 23]. In contrast, the limited responsiveness observed in our study points toward a subgroup with entrenched views, requiring tailored strategies beyond standard information campaigns.

Compared with studies in other European and US contexts, where provider recommendation and school mandates have shown stronger influence [16, 21, 24], the low conditional acceptance observed here suggests deeper ideological or informational resistance. This aligns with systematic reviews reporting that provider trust and social norms are among the most important modifiable factors influencing parental vaccine hesitancy. Importantly, these findings highlight the need to distinguish between “fence-sitters,” who may be influenced by physician advice or peer endorsement [21], and a smaller group of firmly resistant parents, for whom traditional informational campaigns are unlikely to be effective. For these parents, resources should instead focus on strengthening provider–parent communication and targeted interventions that leverage trusted relationships. The dyadic analysis highlights unique gender effects that extend beyond the sex of the parent alone. Parent–child relational patterns appear relevant for understanding how vaccination decisions are negotiated within families. To our knowledge, this perspective is novel in the German context and can guide the design of gender-sensitive communication strategies. Placing our findings in an international context emphasizes both commonalities and unique contributions. Similar drivers of HPV vaccination uptake—knowledge, fear of disease, and healthcare provider influence—have been reported worldwide. However, our dyadic analysis adds a novel perspective by explicitly examining parent–child gender interactions, an area rarely addressed in global literature. This approach complements existing international evidence and highlights the need for gender-sensitive interventions that are adaptable to local cultural and structural conditions [5, 18, 19].

### Strengths and limitations

This study has several strengths, including a relatively large sample size, detailed analysis of parent–child dyads, and the use of multivariable modeling to identify determinants of willingness to vaccinate. To our knowledge, this is the first study in Germany to explore dyadic interactions between parent and child gender in relation to HPV vaccination attitudes.

Nevertheless, important limitations must be acknowledged. Only 4 of the 12 contacted schools participated, which may have introduced selection bias and limits the representativeness of the sample. The sample overrepresented mothers and individuals with higher education, which likely leads to an overestimation of awareness and willingness compared to the general parent population, and therefore our results may represent an optimistic scenario rather than the true situation in Saxony-Anhalt. All data were self-reported and vaccination status was not independently verified, which may have introduced reporting bias. Furthermore, the cross-sectional design precludes conclusions about causality, and results may not be generalizable beyond the Magdeburg region.

Despite these limitations, the study highlights critical gendered dynamics in HPV vaccination acceptance and identifies modifiable determinants that can inform more targeted interventions.

### Conclusion and implications

Efforts to improve HPV vaccination uptake in Saxony-Anhalt should aim to engage all parent groups, including fathers, who in this study demonstrated lower awareness and willingness compared to mothers. Communication strategies that explicitly address the relevance of vaccination for boys and correct gendered misconceptions may be particularly valuable. Strengthening paediatric counselling and providing clear, accessible information targeting both cognitive (knowledge) and emotional (perceived severity) drivers may help translate awareness into acceptance.

A substantial proportion of refusing parents reported that no listed condition would change their decision, suggesting a subgroup with firm prior attitudes distinct from undecided or ambivalent individuals described in vaccine behaviour frameworks. Addressing this subgroup may require tailored approaches beyond standard information campaigns, including trust-building interventions and consistent long-term communication.

These findings may inform local and national HPV vaccination strategies and support Germany’s progress within the WHO Cervical Cancer Elimination Initiative, while contributing to the broader evidence base on gendered patterns in vaccine behaviour.

Future research should explore whether these gendered patterns persist across regions and whether school-based or system-level delivery models could reduce parental variation in HPV vaccine acceptance.

## Supplementary Information

Supplementary Material 1.

Supplementary Material 2.

## Data Availability

The datasets generated and analyzed during the current study are not publicly available due to data protection regulations but are available from the corresponding author upon reasonable request.

## References

[1] Schiffman M, Castle PE, Jeronimo J, Rodriguez AC, Wacholder S. Human papillomavirus and cervical cancer. Lancet. 2007;370(9590):890–907. 17826171 10.1016/S0140-6736(07)61416-0

[2] Robert KI. HPV-Impfstatus Bei Jugendlichen in deutschland: ergebnisse der schuleingangsuntersuchung 2021. Epidemiologisches Bull. 2022;45:3–12.

[3] Bade V, Schmitz H, Tawiah BB. Regional variations in vaccination against COVID-19 in Germany. PLoS ONE. 2024;19(4):e0296976. 38635523 10.1371/journal.pone.0296976 PMC11025766

[4] Bartig S, Muters S, Hoebel J, Schmid-Kupke NK, Allen J, Hovener C. Social differences in COVID-19 vaccination status - Results of the GEDA 2021 study. J Health Monit. 2023;8(Suppl 2):2–22. 37152442 10.25646/11268 PMC10155233

[5] Heyde S. Global parental acceptance, attitudes, and knowledge related to HPV vaccinations for their children: A systematic review and meta-analysis. BMC Women’s Health; 2024. 10.1186/s12905-024-03377-5 PMC11428909 39334328

[6] Achimaș-Cadariu T. Vaccine Hesitancy among European Parents: Socio-demographic and Psychological Factors. Vaccines. 2024. 10.3390/vaccines12020127 PMC10891840 38400111

[7] Chandeying N, Thongseiratch T. Systematic review and meta-analysis comparing educational and reminder digital interventions for promoting HPV vaccination uptake. Npj Digit Med. 2023;6(1):162. 37644090 10.1038/s41746-023-00912-w PMC10465590

[8] Escoffery C, Petagna C, Agnone C, Perez S, Saber LB, Ryan G, et al. A systematic review of interventions to promote HPV vaccination globally. BMC Public Health. 2023;23(1):1262. 37386430 10.1186/s12889-023-15876-5 PMC10308645

[9] Tobaiqy M, MacLure K. A systematic review of human papillomavirus vaccination challenges and strategies to enhance uptake. Vaccines. 2024;12(7):746. 10.3390/vaccines12070746 PMC11281456 39066384

[10] Dempsey AF, Zimet GD, Davis RL, Koutsky L. Factors that are associated with parental acceptance of human papillomavirus vaccines: A randomized intervention study of written information about HPV. Cancer Epidemiol Biomarkers Prev. 2006;15(5):1025–32. 10.1542/peds.2005-1381 16651301

[11] Holman DM, Benard V, Roland KB, Watson M, Liddon N, Stokley S. Barriers to human papillomavirus vaccination among US adolescents: A systematic review of the literature. JAMA Pediatr. 2014;168(1):76–82. 24276343 10.1001/jamapediatrics.2013.2752 PMC4538997

[12] Marlow LAV, Forster AS, Wardle J, Waller J. Mothers’ and fathers’ beliefs about HPV vaccination in the UK. Vaccine. 2013;31(37):4996–5002.

[13] Nguyen M, Buhagiar K, Gatt M. Factors influencing parental vaccine hesitancy in europe: A systematic review. Vaccines. 2023;11(2):351.36851232

[14] Oudin Doglioni D, Gauchet A, Gagneux-Brunon A, Bruel S, Banaszuk AS, Thilly N, et al. Shared human papillomavirus vaccine readiness within families: A psychometric analysis of parent-adolescent dyads in France. Health Psychol. 2024;43(12):893–903. 39172389 10.1037/hea0001387

[15] Oudin Doglioni D, Gagneux-Brunon A, Gauchet A, Bruel S, Olivier C, Pellissier G, et al. Psychometric validation of a 7 C-model of antecedents of vaccine acceptance among healthcare workers, parents and adolescents in France. Sci Rep. 2023;13(1):19895. 37963903 10.1038/s41598-023-46864-9 PMC10646074

[16] Gilkey MB, Calo WA, Marciniak MW, Brewer NT. Parents who refuse or delay HPV vaccine: differences in vaccination behavior, beliefs, and clinical communication preferences. Pediatrics. 2016;138(1):e20153127. 10.1080/21645515.2016.1247134 PMC5360115 27763818

[17] Mavundza EJ. Factors associated with HPV vaccine acceptance and uptake among adolescents and young adults globally. Front Public Health. 2024;14(11):e082592. 10.1136/bmjopen-2023-082592 PMC11575253 39542479

[18] Ntonifor MMN. Factors associated with parental hesitancy towards the human papillomavirus vaccine: a cross-sectional study in Cameroon. Sci Rep. 2025;15(1):18284. 10.1038/s41598-025-94067-1 PMC12104384 40415103

[19] Brunton CGA, Cross-Cultural. Qualitative analysis in Scotland. Spain… Int J Behav Med. 2025. 10.1007/s12529-025-10387-6. 10.1007/s12529-025-10387-6 PMC13623987 40775576

[20] López N. HPV knowledge and vaccine acceptance among European parents in gender-neutral, free-of-charge, school-based vaccination settings. BMC Public Health. 2020;41:10.

[21] Malo TL, Giuliano AR, Kahn JA, Zimet GD, Lee JH, Zhao X. Physicians’ human papillomavirus vaccine recommendations in the context of permissive guidelines for male patients: A National study. Hum Vaccin Immunother. 2014;10(10):2889–96. 10.1158/1055-9965.EPI-14-0344 PMC4184998 25028456

[22] Shapiro GK. The impact of publicly funded immunization programs on HPV vaccine uptake in boys when school-based or government-funded programs are introduced. Lancet Regional Health – Americas; 2022. 10.1016/j.lana.2021.100128 PMC9904075 36778727

[23] Chan DNS. Factors affecting HPV vaccine uptake among ethnic minority adolescent girls: a systematic review. Cancer Epidemiol. 2023;10(9):100279. 10.1016/j.apjon.2023.100279 PMC10471936 37661962

[24] Perkins RB, Zisblatt L, Legler A, Trucks E, Hanchate A, Gorin S. Effectiveness of a provider-focused intervention to improve HPV vaccination rates in boys and girls. J Community Health. 2023;48(1):142–9. 10.1016/j.vaccine.2014.11.021 25448095

