# Supplementary material for: HPV vaccination hesitancy and acceptance among parents in Saxony-Anhalt, Germany: the role of gender, awareness and fear
Source: BMC Public Health. 2025 Dec 23;26:327. doi: 10.1186/s12889-025-26061-1 (PMC12838413; doi:10.1186/s12889-025-26061-1)
Supplement: Supplementary file 1 — Supplementary Material 1. [file 12889_2025_26061_MOESM1_ESM.docx]

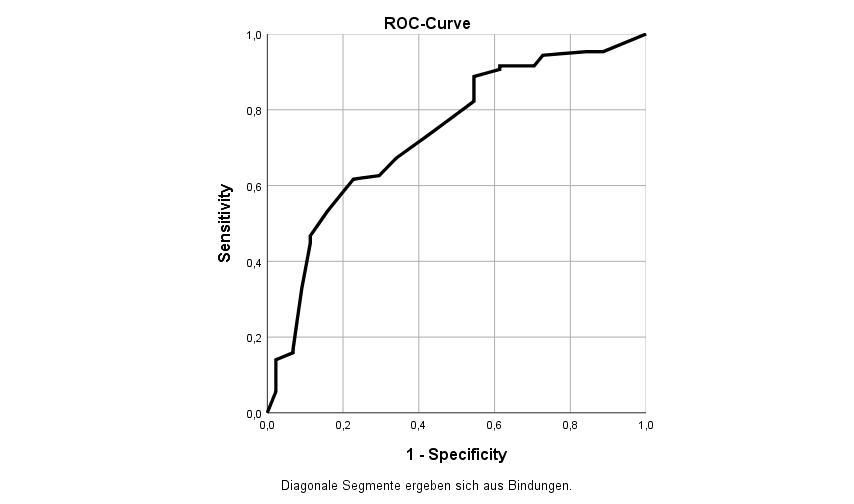


Figure 1. ROC curve for the logistic regression model predicting willingness to vaccinate. AUC = 0.736, indicating acceptable model discrimination.
