# Supplementary material for: HPV vaccination hesitancy and acceptance among parents in Saxony-Anhalt, Germany: the role of gender, awareness and fear
Source: BMC Public Health. 2025 Dec 23;26:327. doi: 10.1186/s12889-025-26061-1 (PMC12838413; doi:10.1186/s12889-025-26061-1)
Supplement: Supplementary file 2 — Supplementary Material 2. [file 12889_2025_26061_MOESM2_ESM.docx]

# Supplementary File 1

## Questionnaire on HPV Vaccination

## Part 1: Personal Information

A1. How old are you?
A2. How old is your child? (9, 10, 11, 12, 13, 14)
A3. What is the gender of your child? (Female, Male)
A4. What is your gender? (Female, Male)
A5. What is your marital status? (Married, Registered partnership, Single, Widowed, Divorced)
A6. What is your highest educational qualification? (Lower secondary education, Intermediate secondary education, University entrance qualification (Abitur), Vocational training / University degree, No response)
A7. What best describes your employment sector? (Industrial/production work, Service sector, Healthcare/social services, Office/administration, Unemployed)
A8. What is your approximate annual gross household income? (≤ €20,000, €20,000–40,000, €40,000–60,000, €60,000–100,000, > €100,000, No response)

## Part 2: HPV and Vaccination

B1. Before this study, had you ever heard of HPV? (Yes / No)
B2. Before this study, had you heard of cervical cancer, anal cancer, or genital warts? (Yes / No)
D3. Are you afraid that you or your child might develop cervical cancer, anal cancer, or genital warts? (Yes / No)
B4. Before this study, had you heard of the HPV vaccine (“cervical cancer vaccine”)? (Yes / No)
B5. Has the mother/legal guardian of the child received HPV vaccination? (Yes / No)
B6. Have you ever actively sought information about HPV vaccination? (Yes / No)
B7. Has your child already been vaccinated against HPV? (Yes / No)
B8. Would you have your child vaccinated against HPV? (Yes / No)
B9. What are your reasons for rejecting HPV vaccination? (Multiple answers possible) [Doubts about vaccine safety; Doubts about vaccine effectiveness; Doubts about the source of the vaccine; Children should decide for themselves; Children are too young for vaccination; None of the above]
B10. Under what conditions would you agree to your child’s HPV vaccination? (Multiple answers possible) [If most of the child’s friends are vaccinated; Strong recommendation by friends/relatives; Family members are vaccinated; Strong recommendation by the child’s doctor; None of the above]
B11. When should HPV vaccination be given? (Before first sexual intercourse; After first sexual intercourse; Don’t know)
B12. At what age should HPV vaccination ideally start? (Younger than 9; 9–11; 12–14; 15–17; Older than 17; Don’t know)
B13. At what stage of schooling should education about HPV vaccination take place? (Primary school; Secondary school; Upper secondary school; University/vocational training; Don’t know; Education about HPV vaccination should not take place)
B14. What is your opinion about future promotion of HPV vaccination in Germany? (In favor – I want HPV vaccination to be promoted; Neutral; Against – I do not want HPV vaccination to be promoted)
B15. If you are against promotion of HPV vaccination, what are your reasons? (Multiple answers possible) [Vaccination could encourage earlier sexual activity among adolescents; More time is needed to study long-term effects; State promotion of the vaccine mainly benefits the pharmaceutical industry; Promotion is interference in a personal decision concerning my child; Other reasons]
